# Bipartite networks represent causality better than simple networks: evidence, algorithms, and applications

**DOI:** 10.3389/fgene.2024.1371607

**Published:** 2024-05-09

**Authors:** Bingran Shen, Gloria M. Coruzzi, Dennis Shasha

**Affiliations:** ^1^ Courant Institute of Mathematical Sciences, Department of Computer Science, New York University, New York, United States; ^2^ Center for Genomics and Systems Biology, Department of Biology, New York University, New York, United States

**Keywords:** RNA sequencing, gene regulatory network, causal inference, random forest, bipartite network

## Abstract

A network, whose nodes are genes and whose directed edges represent positive or negative influences of a regulatory gene and its targets, is often used as a representation of causality. To infer a network, researchers often develop a machine learning model and then evaluate the model based on its match with experimentally verified “gold standard” edges. The desired result of such a model is a network that may extend the gold standard edges. Since networks are a form of visual representation, one can compare their utility with architectural or machine blueprints. Blueprints are clearly useful because they provide precise guidance to builders in construction. If the primary role of gene regulatory networks is to characterize causality, then such networks should be good tools of prediction because prediction is the actionable benefit of knowing causality. But are they? In this paper, we compare prediction quality based on “gold standard” regulatory edges from previous experimental work with non-linear models inferred from time series data across four different species. We show that the same non-linear machine learning models have better predictive performance, with improvements from 5.3% to 25.3% in terms of the reduction in the root mean square error (RMSE) compared with the same models based on the gold standard edges. Having established that networks fail to characterize causality properly, we suggest that causality research should focus on four goals: (i) predictive accuracy; (ii) a parsimonious enumeration of predictive regulatory genes for each target gene *g*; (iii) the identification of disjoint sets of predictive regulatory genes for each target *g* of roughly equal accuracy; and (iv) the construction of a bipartite network (whose node types are genes and models) representation of causality. We provide algorithms for all goals.

## 1 Background and motivation

A frequent goal of expression-based causality research is to construct a directed graph of genes having some inductive and some repressive edges. One of the popular approaches to solve the gene regulatory inference problem is to build some kind of regression models to fit the gene expression data and make regulatory relation inference based on the model parameters. Some of these regression-based methods use linear regression like TIGRESS (Haury et al., 2012), SCODE (Matsumoto et al., 2017), and Inferelator (Skok Gibbs et al., 2022), while others like GENIE3 (Huynh-Thu et al., 2010), BiXGBoost (Zheng et al., 2019), OutPredict (Cirrone et al., 2020), and SCENIC (Van de Sande et al., 2020) choose non-linear regression models for the same goal. The main metric of these methods is conformance to some gold standard (GS) network. However, let us consider the actionable result of such research: to influence the behavior of an organism to make it more useful (e.g., more drought-tolerant crop or one with higher nutrient yield).

This paper asks the question “Are networks a good representation for actionable insights?” Because the edges are simple edges between pairs of regulatory and target genes, the network representation does not suggest any kind of synergy between the putative causal regulatory genes, e.g., transcription factors. So, the natural model choice for a given target gene *g* given the network is a linear model on the genes pointing to *g*.

Any causality model should be able to make reasonably accurate predictions. In Newtonian mechanics, e.g., a model involving mass and gravity will be able to predict the speed curve of a ball on an inclined plane. Predictive accuracy is not **sufficient** to establish causality. Some mysterious force might cause the ball to move in that way, but a causal model should be predictive. We consider predictive accuracy to be a **necessary** condition of a causal model.

We now consider several approaches to prediction.

1. Starting with all transcription factors (TFs) as possible causal features, both a non-linear random forest (RF)-style model *M*_*nonlin*_ and a linear model *M*_*lin*_ are tried.
2. Based on the random forest model mentioned above, a minimal set of TFs that could produce similar regression results in a model *M*_*minimal*_ are iteratively searched for.
3. On the edges of a gold standard network for some target gene *g*, a non-linear model *M*_*nonlin*_ or a linear model *M*_*lin*_ is used.
4. A random forest model that uses the same number of TFs for each target *g* is formed as known from the gold standard network. Those TFs are chosen according to their feature importance starting from *M*_*nonlin*_ in approach 1 above.

As discussed later, (i) the non-linear models work better than the linear models and (ii) starting with all transcription factors and then shrinking that set based on model accuracy is better than using the gold standard network.

Our models are all based on time series in which we predict the mRNA expression level of the target gene based on the state of regulatory genes at the previous time point. This agrees with the biological intuition that the state of causal regulatory genes takes at least minutes to affect their targets. One implication of this approach to model building is that if a transcription factor *T* and a target gene *g* are correlated (e.g., they rise in the same time points and fall in the same time points), *T* will **not** be identified as causal. On the other hand, if *T* rising (respectively, falling) at one time point were associated with *g* rising (respectively, falling) at the next time point, then causality might be hypothesized.

### 1.1 Contributions

Our novel contribution is a framework, algorithms, and software for encoding possible causality in transcriptional settings into a bipartite directed graph. Our framework consists of the following workflow:

1. A machine learning method *M* that predicts the behavior of a target gene *g* starting with all possible causal regulatory elements (transcription factors for genomic networks) as candidates is chosen. *M* may be statistical, a neural network, a forest, or a linear model. We do not advocate any particular model, although non-linear models generally have lower errors than linear ones.
2. The set of possible causal regulatory elements for *g* is reduced to a smaller set *S*_1_ that provides statistically equal (based on the *p*-value) accuracy still based on *M*.
3. Inspired by an observation of the statistician Efron (2020), a set *D* of mutually disjoint sets *S*_1_, *S*_2_ … *S*_*n*_ is found that all provide statistically the same accuracy as *S*_1_ in predicting the expression of some target gene *g*, possibly by training a new model for each *S*_*i*_. *D* may contain *S*_1_ alone or may contain many sets.
4. A visual representation of these mutually disjoint subsets of regulatory elements is provided for each given target gene *g*. The visualization consists of a bipartite graph in which each transcription factor of the disjoint subsets (*S*_1_, *S*_2_ … *S*_*n*_) of transcription factors feeds a model node whose output is the target gene *g*.

## 2 Materials and methods

### 2.1 Expression prediction setup

All the experiments carried out in this study focus on time series RNA sequencing (RNA-seq) data because gene regulation through transcription factors is a temporal causal process. Following this logic, we build regression models that predict the expression of each target gene based on the TF expression levels from a previous time point in the time series. Formally, suppose we are given time series RNA-seq data consisting of sequencing data from time points *t*_0_, *t*_1_, *t*_2_, …., *t*_*i*_, … , *t*_*n*_. We use RNA-seq data at time *t*_*i*_ to predict the expression of a target gene at *t*_*i*+1_ (*i* ≥ 0, *i* < *n*).

In order to split the whole time series into training/testing sets for validating the prediction quality of different methods, we chose to always reserve the tail end of the time series for testing while using the preceding part in training. More specifically, if *n* < 5, then only *t*_*n*_ will be used as the test sample with *t*_*n*−1_ being the input. If 5 ≤ *n* < 10, *t*_*n*_ and *t*_*n*−1_ are reserved for testing, with *t*_*n*−1_ and *t*_*n*−2_ as model input. If *n* ≥ 10, then the final three in the series constitute the testing set.

### 2.2 RNA sequencing data used

Bulk time series RNA-seq data from four different species with varying experimental setups were used, totaling more than 100 data points for each species. The experimental sources for each species are given here. For the training/testing setups below, we train on a prefix of the time points and test on the remaining time points.

1. *Saccharomyces cerevisiae* (yeast): data from GSE145936 (Feder et al., 2021), GSE153609 (Mitra et al., 2021; Tran et al., 2021), GSE168699 (Li et al., 2021), and GSE226769 (Harris and Ünal, 2023) were aggregated into a gene expression dataset with 144 training samples and 58 testing samples.
2. *Bacillus subtilis* (strain 168) (*B. subtilis*): data from GSE108659 (Krawczyk et al., 2015), GSE128875 (Pisithkul et al., 2019), and GSE224332 were aggregated into a gene expression dataset with 84 training samples and 18 testing samples.
3. *Arabidopsis thaliana* (*Arabidopsis*): data from GSE97500 (Varala et al., 2018; Heerah et al., 2021) were used with 72 training samples and 24 testing samples.
4. *Mus musculus* (mouse): data from GSE115553 (Graham et al., 2018), GSE151173 (Greenwell et al., 2020), and GSE171975 (Aviram et al., 2021) were aggregated into a gene expression dataset with 208 training samples and 121 testing samples.
5. *Homo sapiens* (human): data from GSE221103 and GSE221173 (Cazarin et al., 2023) were aggregated into a gene expression dataset consisting of 109 training samples and 40 testing samples.

Thus, our study derives from 4 well-studied living organisms ranging from bacteria to humans. We sought datasets having time series RNA-seq data with relatively tight timing intervals (no greater than 4 h in all cases) and suitably long series (≥4 time points) to form training/testing splits. We collected as much public bulk RNA-seq data about the four species as possible given the above constraints. Because the data came from widely different species (from bacteria to human), we expect that our qualitative conclusions will be generalizable. For each species, we obtained a GS regulatory network from the sources given in Table 1.

**TABLE 1:** Information on the gold standard (GS) networks used in this study. The number of target genes and transcription factors are for genes that are both present in the regulatory network and the RNA-seq data for each species, respectively.

Species	GS network **source**	Number of **target genes**	Number of **TFs**	Number of **regulations**
Yeast	YEASTRACT (Teixeira et al., 2023)	4,794	213	162,100
*B. subtilis*	SubtiWiki (Pedreira et al., 2022)	1,878	146	3,973
*Arabidopsis*	ConnecTF (Brooks et al., 2021)	18,855	57	141,445
Mice	RegNetwork (Liu et al., 2015)	8,211	780	40,331
Humans	RegNetwork (Liu et al., 2015)	17,533	1,351	132,259

### 2.3 Metrics

Because RNA-seq counts are strongly dependent on the amount of cellular material that is read, relative expression is a better metric to determine induction or repression than absolute expression. For that reason, we measure expression based on the z-score of the normalized RNA-seq counts in the form of transcripts per kilobase million (TPM):

$$z=\frac{TPM-\mu }{\sigma }.$$ (1)

To compare the performance of each method, we measure how accurate the regression results were by checking the error of the prediction on the test set for each of the target genes. More specifically, the root mean square error (RMSE) of the model prediction in the test set for the expression of each target gene was compared across different regression models. Because every regression model was trained/fitted to make predictions on the same set of time series expression samples for each target gene in question, the performance metrics can be compared based on a paired test. For this purpose, we use a non-parametric paired test (Katari et al., 2021).

### 2.4 Methods compared

For the purpose of predicting the expression of each target gene on a future unseen time point, we fitted four different types of regression models, as described above:

1. An RF model that takes the expression of all TFs as input.
2. A ridge regression (linear regression with L2 regularization) model that takes the expression of all TFs as input.
3. A random forest model that takes only the expression of TFs known from the GS network for each particular target gene as input.
4. A ridge regression model that takes only the expression of TFs known from the GS network for each particular target gene as input.

Next, we test how good the transcription factors from the GS network are compared to the same number of transcription factors derived from a non-linear model. For each target gene *g*, let *k*_*g*_ be the number of transcription factors in the GS network that point to *g*. In addition to the tests above, we compare a random forest on those GS TFs against a random forest for *g* based on the top *k*_*g*_ TFs found using method 1 above. The idea is to test the usefulness of GS edges for prediction. One may argue that GS edges are inferred using different methods—usually by modifying single regulatory genes and observing their effect—and, therefore, should not necessarily be useful for prediction but could still be useful if modifying a single gene is all that is possible for practical reasons. We do not contest their utility for such purposes. We do, however, aim to evaluate their predictive power in a synergistic setting (i.e., when potentially several regulatory genes can be simultaneously modified).

Finally, using the method given in Section 3, we construct a minimal random forest model for each target gene *g* on the training set and view its result on the test set. We choose random forest because decision tree-based regression models have proven to be among the best methods in gene regulatory tasks (Huynh-Thu et al., 2010; Moerman et al., 2019; Zheng et al., 2019). We did not expand our model selection because the main focus of our work is not to find the best fitted machine learning model for the task but rather to demonstrate a novel approach to the representation of potential causality in gene regulation.

## 3 Algorithms to construct bipartite causality graphs

Having chosen prediction as the metric for causality, we now turn to the other three goals of our proposed framework:

1. Finding minimal sets of predictive TFs.
2. Finding disjoint minimal sets that have *p*-value-indistinguishable predictive accuracy.
3. Creating a bipartite visual representation of causality.

### 3.1 Minimal sets of predictive transcription factors

Efron (2020) noted that disjoint sets of causal factors often have similar predictive accuracy. Inspired by this observation, we propose the following strategy. For a given target gene, a random forest predictor that takes the expression levels of all known TFs is fit to predict the expression of the target. Then, the number of TFs are iteratively cut in half based on their feature importance in the fitted model until a further reduction results in a statistically significantly (*p*-value <0.05 ) worse-performing random forest. We refer to the final remaining set of TFs as the “minimal TF set per target.” The pseudo-code for this feature selection process is shown in Algorithm 1.

**FIGURE 1:**
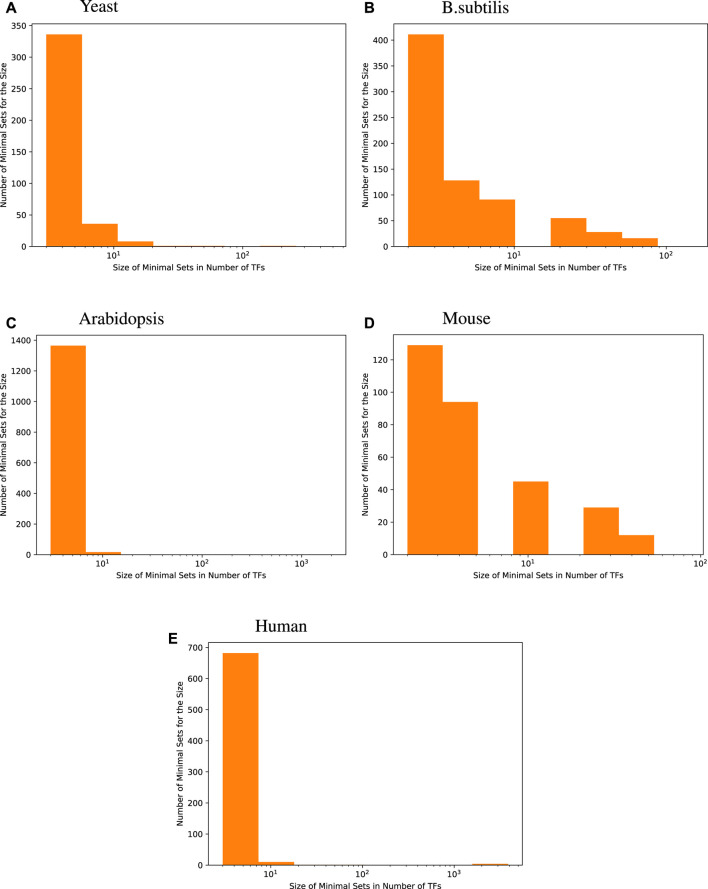
Size distribution of a minimal transcription factor (TF) set for each target gene. For every species, the majority of genes are best predicted by fewer than 8 transcription factors, i.e., 96.6% for yeast, 73.5% for *B. subtilis*, 98.7% for *Arabidopsis*, 71.9% for mice, and 97.7% for humans.

The histograms given in Figure 1 show the distribution of the size of minimal TF sets yielded for each target gene for the four species we investigated. These size distributions show that most of the minimal sets consist of a rather small number of TFs. When compared with the distribution of GS network coverage for each target gene in Table 2, we see that an accurate regression model constructed this way usually has fewer input transcription factors compared to the GS networks.

**TABLE 2:** Gold standard network edge coverage per target gene for different RNA-seq species compared with the size of minimal transcription factor set sizes per target gene, derived using random forest regression. Distributions are presented as the median followed by the interquartile range (IQR), which is the range between the 25th and 75th percentile of the data. The one exception is yeast, where the gold standard edges are much more numerous. As shown in the tables above, the minimal sets generally have better predictive power than the gold standard sets and roughly the same number of edges per target.

Median [IQR]	Yeast	*B. subtilis*	*Arabidopsis*	Mice	Humans
Size of the TF set per target in the gold standard network	51 [41, 63]	1 [1, 2]	13 [6, 19]	4 [2, 7]	8 [4, 14]
Size of the minimal TF set per target using RF regression	3 [3, 3]	2 [2, 9]	3 [3, 3]	5 [2, 10]	3 [3, 3]
**Number of target genes**	385	733	1,373	310	698

Algorithm 1 Minimal TF set per target: For each target gene *G*, initial set of TFs, and RMSE *E*, the set of necessary transcription factors are repeatedly reduced by half until the error increases significantly with respect to *E*. A minimal set of TFs for a given target gene *G* will be a call to this function MinimalSet(*G*, *all TFs*, 0).

1: **function** MinimalSet(*G*, *TFs*, *E*)
2:   *F* ← *TFs*
3:   *M*_*all*_ ← initialized regression model
4:   Fit *M*_*all*_ with *F* to predict *G*
5:   **if** *E* > 0 **then**
6:    *E*_*baseline*_ ← *E*
7:   **else**⊳ *E* == 0 implies that no error value has been calculated yet
8:    *E*_*baseline*_ ← training Error of *M*_*all*_
9:   *F*_*half*_ ← top half most influential TFs used in *M*_*all*_
10:   *flag* ← *True*
11:   **while** *flag* == *True* **do**
12:    *M*_*current*_ ← initialized regression model
13:    Fit *M*_*current*_ with *F*_*half*_ to predict target gene *G*
14:    *E*_*current*_ ← training Error of *M*_*current*_
15:    **if not** *E*_*current*_ > *E*_*baseline*_ with statistical significance **then**
16:     *F* ← *F*_*half*_
17:     *F*_*half*_ ← top half most influential TFs used in *M*_*current*_
18:    **else**
19:     *flag* ← *False*
20:   **return** *F*

### 3.2 Finding disjoint sets of predictive transaction factors

After finding a minimal set of predictive TFs, our algorithm performs a new round of TF searches to discover disjoint sets of roughly equally predictive TFs. Algorithm 2 describes the process for finding all such disjoint sets of a given target gene. Similar to the minimal set search algorithm, we based our iterative search on the random forest regression that takes all available TFs *U* as input. Rather than stopping after a minimal set *S*1 is found, we test if using all remaining TFs (*U* − *S*1) could also produce a regression prediction as good as the baseline. If that is the case, we carry on a similar feature reduction process that ends with a new “minimal set” *S*2 from *U* − *S*1. This process then repeats with *U* − (*S*1 ∪ *S*2) and continues until the baseline performance cannot be beaten or there are no TFs left. For each target gene *g*, we define the collection of minimal sets discovered this way as the *minimal disjoint sets of predictive transcription factors for* *g* or *MinDisjoints(* *g* *)* for short.

**FIGURE 2:**
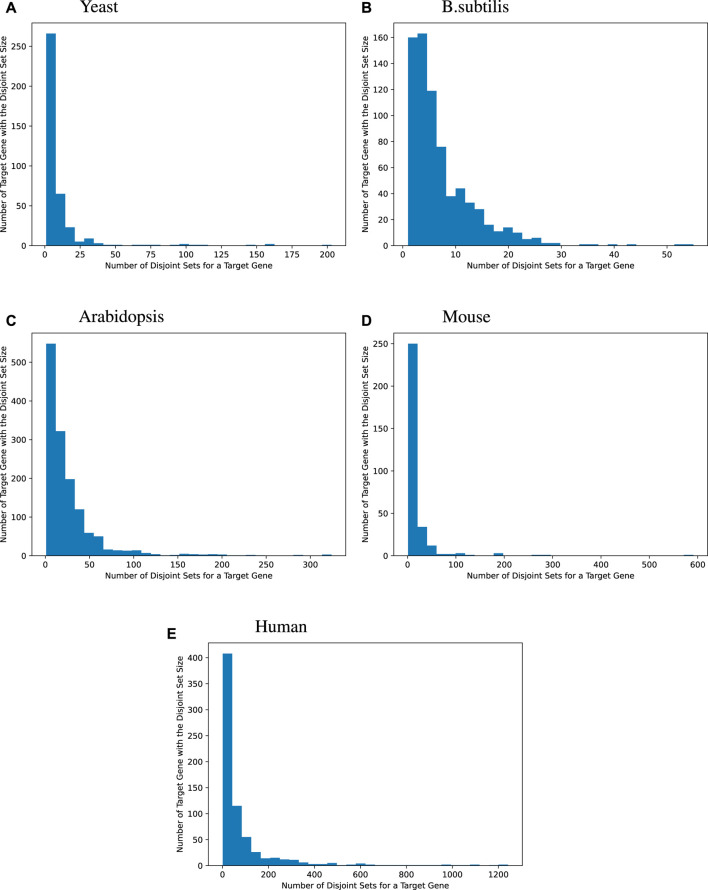
Distribution of the disjoint set count for each target gene. Many target genes can be best modeled by a handful of an explanatory set of TFs. For yeast, 44.2% of the target genes are best modeled by fewer than 5 explanatory disjoint sets of TFs, For *B. subtilis*, 44.1%, for *Arabidopsis*, 15.2%, for mice, 25.5%, and for humans, 10.3%. Sometimes, many disjoint sets of TFs are redundant. Bipartite graphs capture this causality information.

We then surveyed the distribution of how many MinDisjoints are found for each target gene across the four species. The histograms given in Figure 2 show that most of the target genes have a small number of disjoint sets of TFs associated with them, while some target genes have a large number of MinDisjoints. Our analysis did not yield biological mechanisms for predictability/causality, so we have no mechanistic explanation for how multiple disjoint sets of TFs might control the same target gene. However, the result was not wholly unexpected, given the well-known redundancy in biological systems.

Algorithm 2. Disjoint sets of TFs: Finding minimal sets of disjoint TFs (MinDisjoints), where each minimal set has the same error as using all TFs.

1:  *D* ← empty list
2:  *F* ← All TFs
3:  *M*_*all*_ ← initialized regression model
4:  Fit *M*_*all*_ with *F* to predict target gene *G*
5:  *E*_*all*_ ← training Error of *M*_*all*_
6:  *F*_*m*_ ← MinimalSet(*G*, *F*, *E*_*all*_)
7:  Add *F*_*m*_ to *D*
8:  *F*_*r*_ ← *F* \ *F*_*m*_
9:  *flag* ← *True*
10:  **while** *F*_*r*_ ≠ *Ø* **and** *flag* == *True* **do**
11:   *M*_*r*_ ← initialized regression model
12:   Fit *M*_*r*_ with *F*_*r*_ to predict target gene *G*
13:   *E*_*r*_ ← training Error of *M*_*r*_
14:   **if** *E*_*r*_ > *E*_*all*_, with statistical significance **then**
15:    *flag* ← *False*
16:    **break**
17:   *F* ← MinimalSet(*G*, *F*_*r*_, *E*_*all*_)
18:   Add *F* to *D*
19:   *F*_*r*_ ← *F*_*r*_ \ *F*
20: For a given target gene *G*, *D* will be the set of disjoint sets of TFs *G*.

### 3.3 Bipartite network representation

Networks have a pleasing visual representation, especially when focusing on one or a few target genes. However, what we showed is that the network itself is a poor basis for prediction. Now that we have constructed multiple disjoint sets of predictive TFs for each target gene *g*, we propose a bipartite representation for them. The bipartite representation for each target gene *g* consists of a model node *m* corresponding to each disjoint set *d*_*m*_ from D(*g*). The TFs from *d*_*m*_ in turn point to *m*.

Suppose that TFs A, B, and C through model M(A, B, C) provide good predictions regarding target gene *g*. Suppose further that TFs D, E, F, and H provide roughly equally good predictions on *g*. The classic gene regulatory approach would be a graph with arrows from A, B, C, D, E, F, and H all pointing to *g*. The bipartite approach would suggest instead to show a bipartite graph that would have A, B, and C point to a model node, which, in turn, points to g, and have D, E, F, and H point to a different model node, which also points to g.

**FIGURE 3:**
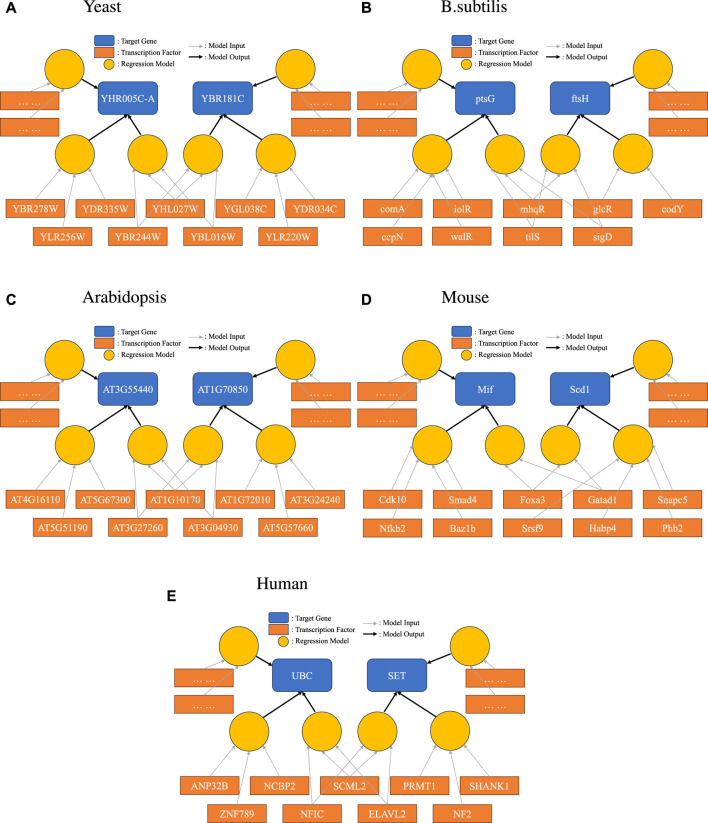
Bipartite representation of causality. Light circular orange nodes represent non-linear models that take transcription factors (dark orange rectangles) as inputs and produce predictions on a single target gene (blue). Here, we show a particular case where disjoint sets of TFs can form high-quality prediction models for one target gene, and the same TF can be in models for several target genes.

To demonstrate this new representation, we picked one example for each species we studied, as shown in Figure 3. Here, we specifically highlighted one interesting scenario: a set of TFs were found to form one of the disjoint sets for more than one target gene. Such a relationship between two genes would not have been found in a simple network representation. The bipartite representation reveals group effects that would not otherwise be evident.

## 4 Results

### 4.1 Comparison of approaches

**FIGURE 4:**
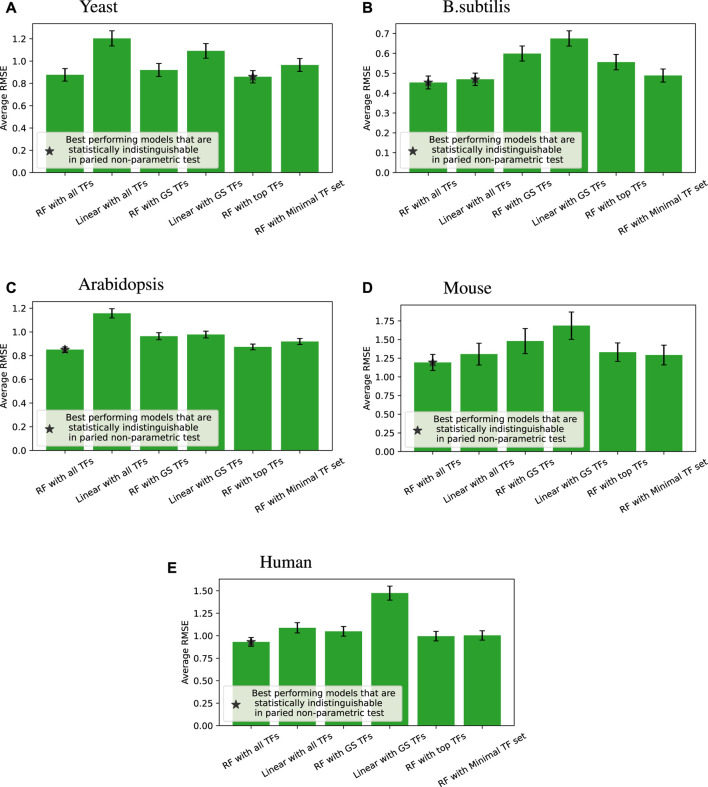
Root mean square error (RMSE) performance (lower is better) of different regression models compared across four species; error bars represent the standard error of each group. When we compare all the models for each of the tested target genes, a paired non-parametric test can be applied between each pair of models to see if the performance is statistically different. The best performing models that are statistically indistinguishable this way are marked with *. “RF with all TFs” is always one of the best performing models. The caption of Table 2 provides a glossary of terms.

Figure 4 shows the accuracy of the six different modeling approaches listed in Section 2.4. Basically, feeding expression information from all the TFs into a random forest (“RF with all TFs”) yielded the best outcome. Relying solely on known GS edges (“RF with GS TFs”) usually performed poorly, even compared to using the same number of TFs for each target gene from the random forest model (“RF with top TFs”).

We note that linear models on all TFs are competitive and sometimes better than random forests on minimal TF sets for *B. subtilis* and mouse. Still, overall, given the same input information, random forests perform better than linear models, which is the main point of that comparison.

Tables 3–7 list the detailed pairwise non-parametric results comparing the performance of all possible pairs of models. The tables show that using all TFs in the regression yields the highest prediction accuracy. Finding a minimal set of the most important TFs yields almost the same accuracy as using all TFs.

**TABLE 3:** ***S. cerevisiae* (yeast):** Paired non-parametric results for the performance comparison on the test set between the model in blue and orange measured using root mean square error (RMSE) on target gene expression in the test datasets. Entry (*i*, *j*) shows the 95% confidence interval as the difference of the *i*th blue modeling method minus the *j*th orange modeling method. A negative number means the method in blue has a lower error and, hence, is better. When the difference in the blue method *i* with the red method *j* has a *p*-value below 0.05 based on a non-parametric paired test, the (*i*, *j*)th confidence interval will be red or blue. Otherwise, the (*i*, *j*)th entry will be black. Glossary: (i) TF = transcription factor; (ii) GS = gold standard; (iii) RF = random forest; (iv) linear = ridge regression; (v) RF with top TFs = for each target gene g, the same number of TFs from the random forest model are used as there were gold standard edges for g; and (vi) minimal TF = minimal set of most important TFs that provides *p*-value-indistinguishable results (on the training set) using all TFs.

	RF with all TFs	Linear with all TFs	RF with GS TFs	Linear with GS TFs	RF with top TFs	RF with the minimal TF set
Mean RMSE	0.877	1.204	0.920	1.091	0.859	0.965
RF with all TFs	-	(−0.397, −0.257)	(−0.055, −0.031)	(−0.263, −0.166)	(0.008, 0.027)	(−0.119, −0.058)
Linear with all TFs	(0.257, 0.397)	-	(0.214, 0.354)	(0.047, 0.178)	(0.275, 0.414)	(0.169, 0.309)
RF with GS TFs	(0.031, 0.055)	(−0.354, −0.214)	-	(−0.218, −0.124)	(0.046, 0.076)	(−0.075, −0.015)
Linear with GS TFs	(0.166, 0.263)	(−0.178, −0.047)	(0.124, 0.218)	-	(0.185, 0.279)	(0.081, 0.172)
RF with top TFs	(−0.027, −0.008)	(−0.414, −0.275)	(−0.076, −0.046)	(−0.279, −0.185)	-	(−0.133, −0.079)
RF with the minimal TF set	(0.058, 0.119)	(−0.309, −0.169)	(0.015, 0.075)	(−0.172, −0.081)	(0.079, 0.133)	-

**TABLE 4:** ***B. subtilis*:** Paired non-parametric results for the performance comparison between the model in blue and orange measured using RMSE on target gene expression in the test datasets. Entry (*i*, *j*) shows the 95% confidence interval as the difference of the *i*th blue modeling method minus the *j*th orange modeling method. A negative number means the method in blue has a lower error and, hence, is better. When the difference in the blue method *i* with the red method *j* has a *p*-value below 0.05 based on a non-parametric paired test, the (*i*, *j*)th confidence interval will be red or blue. Otherwise, the (*i*, *j*)th entry will be black. The caption of Table 2 provides a glossary of terms.

	RF with all TFs	Linear with all TFs	RF with GS TFs	Linear with GS TFs	RF with top TFs	RF with the minimal TF set
Mean RMSE	0.454	0.469	0.599	0.675	0.556	0.489
RF with all TFs	-	(−0.035, 0.003)	(−0.168, −0.122)	(−0.247, −0.196)	(−0.124, −0.080)	(−0.053, −0.017)
Linear with all TFs	(−0.003, 0.035)	-	(−0.161, −0.098)	(−0.243, −0.168)	(−0.113, −0.060)	(−0.037, −0.001)
RF with GS TFs	(0.122, 0.168)	(0.098, 0.161)	-	(−0.099, −0.054)	(0.015, 0.071)	(0.080, 0.141)
Linear with GS TFs	(0.196, 0.247)	(0.168, 0.243)	(0.054, 0.099)	-	(0.086, 0.153)	(0.153, 0.221)
RF with top TFs	(0.080, 0.124)	(0.060, 0.113)	(−0.071, −0.015)	(−0.153, −0.086)	-	(0.044, 0.091)
RF with the minimal TF set	(0.017, 0.053)	(0.001, 0.037)	(−0.141, −0.080)	(−0.221, −0.153)	(−0.091, −0.044)	-

**TABLE 5:** ***Arabidopsis*:** Paired non-parametric results for the performance comparison between the model in blue and orange measured using the RMSE on target gene expression in the test datasets. Entry (*i*, *j*) shows the 95% confidence interval as the difference of the *i*th blue modeling method minus the *j*th orange modeling method. A negative number means the method in blue has a lower error and, hence, is better. When the difference in the blue method *i* with the red method *j* has a *p*-value below 0.05 based on a non-parametric paired test, the (*i*, *j*)th confidence interval will be red or blue. Otherwise, the (*i*, *j*)th entry will be black. The caption of Table 2 provides a glossary of terms.

	RF with all TFs	Linear with all TFs	RF with GS TFs	Linear with GS TFs	RF with top TFs	RF with the minimal TF set
Mean RMSE	0.851	1.157	0.964	0.978	0.874	0.919
RF with all TFs	-	(−0.345, −0.268)	(−0.135, −0.092)	(−0.148, −0.108)	(−0.032, −0.014)	(−0.080, −0.057)
Linear with all TFs	(0.268, 0.345)	-	(0.159, 0.227)	(0.142, 0.215)	(0.245, 0.322)	(0.203, 0.274)
RF with GS TFs	(0.092, 0.135)	(−0.227, −0.159)	-	(−0.028, −0.001)	(0.069, 0.112)	(0.023, 0.067)
Linear with GS TFs	(0.108, 0.148)	(−0.215, −0.142)	(0.001, 0.028)	-	(0.086, 0.124)	(0.039, 0.081)
RF with top TFs	(0.014, 0.032)	(−0.322, −0.245)	(−0.112, −0.069)	(−0.124, −0.086)	-	(−0.057, −0.033)
RF with the minimal TF set	(0.057, 0.080)	(−0.274, −0.203)	(−0.067, −0.023)	(−0.081, −0.039)	(0.033, 0.057)	-

**TABLE 6:** **Mice:** Paired non-parametric results for the performance comparison between the model in blue and orange measured using the RMSE on target gene expression in the test datasets. Entry (*i*, *j*) shows the 95% confidence interval as the difference of the *i*th blue modeling method minus the *j*th orange modeling method. A negative number means the method in blue has a lower error and, hence, is better. When the difference in the blue method *i* with red method *j* has a *p*-value below 0.05 based on a non-parametric paired test, the (*i*, *j*)th confidence interval will be red or blue. Otherwise, the (*i*, *j*)th entry will be black. The caption of Table 2 provides a glossary of terms.

	RF with all TFs	Linear with all TFs	RF with GS TFs	Linear with GS TFs	RF with top TFs	RF with the minimal TF set
Mean RMSE	1.194	1.305	1.480	1.686	1.331	1.293
RF with all TFs	-	(−0.217, −0.006)	(−0.437, −0.136)	(−0.669, −0.316)	(−0.200, −0.074)	(−0.169, −0.029)
Linear with all TFs	(0.006, 0.217)	-	(−0.261, −0.088)	(−0.503, −0.259)	(−0.110, 0.059)	(−0.055, 0.080)
RF with GS TFs	(0.136, 0.437)	(0.088, 0.261)	-	(−0.290, −0.122)	(0.032, 0.266)	(0.072, 0.303)
Linear with GS TFs	(0.316, 0.669)	(0.259, 0.503)	(0.122, 0.290)	-	(0.203, 0.508)	(0.248, 0.539)
RF with top TFs	(0.074, 0.200)	(−0.059, 0.110)	(−0.266, −0.032)	(−0.508, −0.203)	-	(−0.025, 0.101)
RF with the minimal TF set	(0.029, 0.169)	(−0.080, 0.055)	(−0.303, −0.072)	(−0.539, −0.248)	(−0.101, 0.025)	-

**TABLE 7:** **Humans:** Paired non-parametric results for the performance comparison between the model in blue and orange measured using the RMSE on target gene expressions in the test datasets. Entry (*i*, *j*) shows the 95% confidence interval as the difference of the *i*th blue modeling method minus the *j*th orange modeling method. A negative number means the method in blue has a lower error so is better. When the difference in the blue method *i* with red method *j* has a *p*-value below 0.05 based on a non-parametric paired test, the (*i*, *j*)th confidence interval will be red or blue. Otherwise, the (*i*, *j*)th entry will be black. The caption of Table 2 provides a glossary of terms.

	RF with all TFs	Linear with all TFs	RF with GS TFs	Linear with GS TFs	RF with top TFs	RF with the minimal TF set
Mean RMSE	0.931	1.088	1.049	1.474	0.995	1.004
RF with all TFs	-	(−0.192, −0.122)	(−0.143, −0.093)	(−0.616, −0.469)	(−0.081, −0.047)	(−0.088, −0.056)
Linear with all TFs	(0.122, 0.192)	-	(0.005, 0.073)	(−0.457, −0.314)	(0.060, 0.126)	(0.052, 0.117)
RF with GS TFs	(0.093, 0.143)	(−0.073, −0.005)	-	(−0.489, −0.361)	(0.032, 0.076)	(0.019, 0.072)
Linear with GS TFs	(0.469, 0.616)	(0.314, 0.457)	(0.361, 0.489)	-	(0.413, 0.545)	(0.400, 0.541)
RF with top TFs	(0.047, 0.081)	(−0.126, −0.060)	(−0.076, −0.032)	(−0.545, −0.413)	-	(−0.029, 0.012)
RF with the minimal TF set	(0.056, 0.088)	(−0.117, −0.052)	(−0.072, −0.019)	(−0.541, −0.400)	(−0.012, 0.029)	-

A question to ask is what biological meaning disjoint sets of transcription factors could have for a given target gene *g*. Our computational analysis does not provide a biological meaning other than predictive ability. Experimentalists might take various disjoint sets of TFs and manipulate them to achieve some desired effect on a target gene. The choice of such sets may depend on the side effects such manipulation might have on other genes. This is a direction for future work.

### 4.2 Batch effects

While the z-score takes care of quantity bias in different tests, batch effects may cause predictions on batch A based on data from batch A to be superior to predictions on batch A from data on many batches. This is a limitation of any predictive model in biology.

**FIGURE 5:**
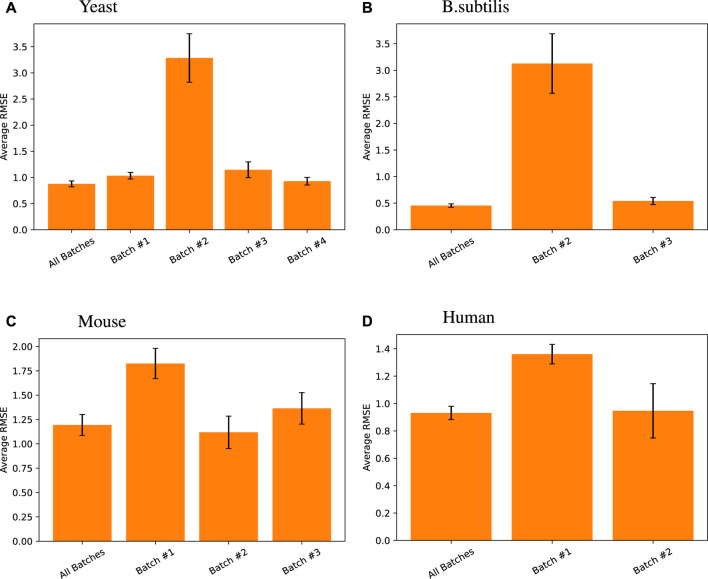
Random forest regression performance differences measured across different data batches for different species. Here, all batches results are default and presented relists shown in this work, where the random forest model using all TFs was trained on training data from all batches and tested on all the tail testing parts from different batches. The same model was then trained and tested on individual batches for its respective high-variance target genes. Note that for *Arabidopsis*, one singular batch was used, so such comparison was unnecessary, and for *B. subtilis*, the first batch does not have high-variance target genes in its testing set, so the comparison was also omitted.

To test this, we created our models based on multiple batches and tested them on the tail end of all those batches. We compared that approach with batch-by-batch predictions. Figure 5 shows that the same model trained on all batches of data achieves the same level or better predictive performance than when using batch X data on batch X tail, for each batch X.

### 4.3 Ensemble of disjoint sets of transcription factors

Another potential use of the identification of disjoint sets of predictive TFs stems from the fact that each disjoint set represents a regression model for the prediction of the target gene. For all the disjoint sets, we found a target gene *g*, and the regression model of each disjoint set can provide a prediction about the expression of the target gene, given the expression input of the TFs at the previous time point. As has been shown in many previous studies in both general machine learning and gene network inference (Dietterich, 2000; Marbach et al., 2012; Sagi and Rokach, 2018; Ganaie et al., 2022), an ensemble consisting of the arithmetic mean of these model predictions may lead to an overall better performing prediction. Inspired by those results, we compared the predictive performance of this ensemble of disjoint sets of TFs to that of all other RF-based regressions we discussed before, and the results are given in Table 8. In most cases, this ensemble yielded regression accuracies second only to the model that takes all TFs as input.

**TABLE 8:** Paired non-parametric results for the performance comparison between the model in blue and orange measured using the RMSE on target gene expressions in the test datasets. Each column is a comparison for one of the four species that compared the ensemble prediction from disjoint sets of transcription factors (TFs) to other random forest (RF)-based methods. Entry (*i*, *j*) shows the 95% confidence interval as the difference of the *i*th blue modeling method minus the *j*th orange modeling method. A negative number means the method in blue has a lower error and, hence, is better. When the difference in the blue method *i* with the red method *j* has a *p*-value below 0.05 based on a non-parametric paired test, the (*i*, *j*)th confidence interval will be red or blue. Otherwise, the (*i*, *j*)th entry will be black. The caption of Table 2 provides a glossary of terms.

	Ensemble of disjoint sets in yeast	Ensemble of disjoint sets in *B. subtilis*	Ensemble of disjoint sets in *Arabidopsis*	Ensemble of disjoint sets in mice	Ensemble of disjoint sets in humans
RF with all TFs	(−0.058, −0.017)	(−0.009, 0.008)	(−0.009, 0.001)	(−0.038, 0.013)	(−0.021, −0.003)
RF with GS TFs	(0.227, 0.366)	(−0.003, 0.034)	(0.270, 0.349)	(0.034, 0.223)	(0.115, 0.181)
RF with top TFs	(−0.010, 0.027)	(0.122, 0.170)	(0.090, 0.133)	(0.169, 0.444)	(0.085, 0.134)
RF with the minimal TF set	(0.138, 0.231)	(0.196, 0.249)	(0.105, 0.146)	(0.350, 0.681)	(0.464, 0.610)

A complete list of all the minimal sets and disjoint sets of TFs for each target gene we surveyed in this study is given in Supplementary Table S1–S5.

### 4.4 An application: optimizing gene expression

Suppose our goal is to cause a gene g to be expressed at a certain level. We observed that the GS network, even when available, provides quite poor predictions. A better approach is to start with a good predictive model for g on a small number of TFs T and then to determine values of the TFs in T that might lead to the desired expression level of g. This goal is supported by the three goals of our framework: to find a good model, reduce the number of TFs while preserving accuracy, and find possible alternative sets of TFs that also yield high prediction accuracy. Gene regulatory networks do not provide natural guidance for any goal like this.

Thus, the bipartite network approach provides an actionable approach to causality. At the same time, it provides (i) a visualization that shows alternative ways to manipulate a target gene and (ii) a simple ensemble approach to prediction.

## 5 Empirical findings

Our empirical findings are as follows:

• We confirm previous observations (Pratapa et al., 2020; Zhao et al., 2021) that non-linear models generally yield better results (as measured by RMSE) than linear models.
• Using all TFs yields better predictive results than using the TFs from GS edges. For each target gene *g*, there often exist several disjoint minimal sets (mostly of size eight or less) that yield predictive accuracy nearly as high as all TFs.
• Using all batches of each species together for training yields results on the time series test tails of each batch that are as good as or better than using each batch on its own test tail.
• For a given target gene *g*, forming a model consisting of the most influential *k*_*g*_ TFs in a non-linear model (e.g., random forests) as measured on the training set, where *k*_*g*_ is the number of TFs in the GS network that point to *g*, yields better prediction accuracy on the test set than using the same kind of model on the GS TFs. This superiority holds for all the species we tested from yeast with a mean value of 53 TFs for each target gene to *B. subtilis* with a mean value of 1.9 TFs for each target gene.

## 6 Conclusion

Based on our empirical findings, we suggest a framework for studying causality in gene regulation having three main features.

First, the figure of merit for causality should be predictive accuracy rather than conformance with “gold standard” edges. One reason is epistemic: any causal model should be predictive. Another reason is pragmatic: prediction is useful if we want to manipulate some property such as the expression of a target gene.

Second, the network representation of such causality should be a bipartite graph consisting of gene (including transcription factor) nodes and model nodes. Such graphs encode the synergy of multiple TFs in the model nodes.

Third, the bipartite representation may include many model nodes that point to the same target gene, where each model node has a disjoint set of TFs as input. A single TF plays a role in disjoint sets of several target genes.

In addition to suggesting a modified approach to causality research for transcriptional regulation, we assert that our framework is applicable beyond transcriptional causality. Our main future work is to apply this form of analysis to other multifactor causality domains. We welcome other researchers to try this approach and offer our software to help.

## Data Availability

The datasets presented in this study can be found in online repositories. The names of the repository/repositories and accession number(s) can be found at: https://github.com/IcyFermion/feature_synergy.
