# Corrigendum: Bipartite networks represent causality better than simple networks: Evidence, algorithms, and applications

**DOI:** 10.3389/fgene.2024.1440665

**Published:** 2024-06-18

**Authors:** Bingran Shen, Gloria M. Coruzzi, Dennis Shasha

**Affiliations:** ^1^ Courant Institute of Mathematical Sciences, Department of Computer Science, New York University, New York, United States; ^2^ Center for Genomics and Systems Biology, Department of Biology, New York University, New York, United States

**Keywords:** RNA sequencing, gene regulatory network, causal inference, random forest, bipartite network

In the published article, an **Author name** was incorrectly written as “Gloria Curozzi.” The corrected author name should read as “Gloria M. Coruzzi.”

The authors apologize for this error and state that this does not change the scientific conclusions of the article in any way. The original article has been updated.

