# Supplementary material for: Bipartite networks represent causality better than simple networks: evidence, algorithms, and applications
Source: Front Genet. 2024 May 9;15:1371607. doi: 10.3389/fgene.2024.1371607 (PMC11120958; doi:10.3389/fgene.2024.1371607)
Supplement: Supplementary file 2 [file Image1.PDF]

Supplementary Material

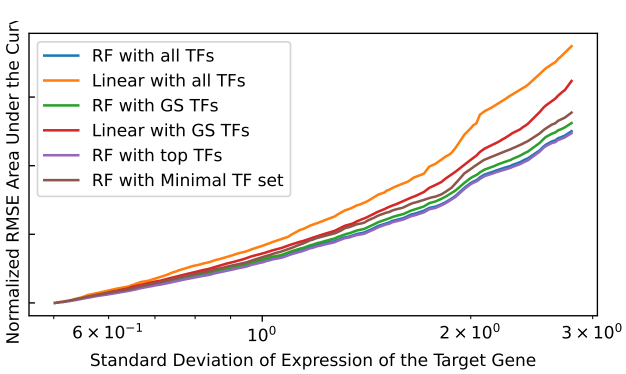

Figure 1a. Yeast

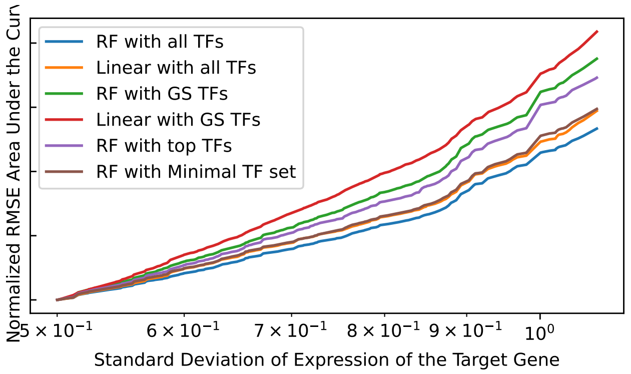

Figure 1b. B.subtilis

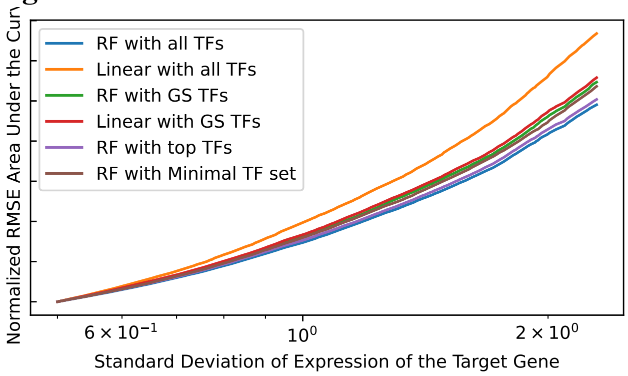

Figure 1c. Arabidopsis

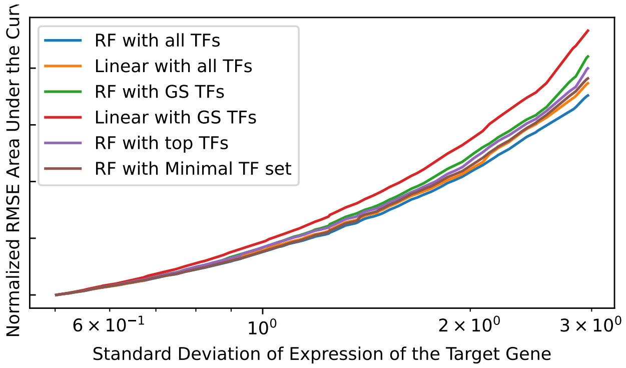

Figure 1d. Mouse

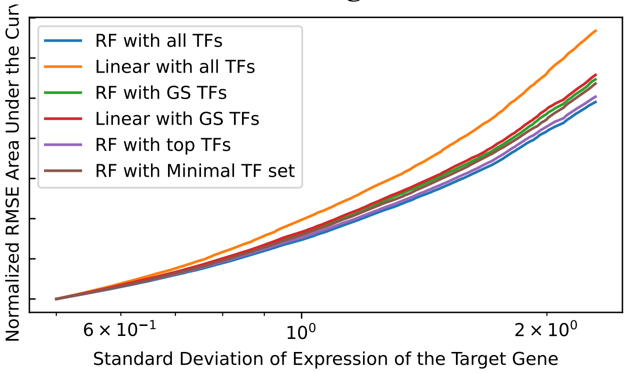

Figure 1e. Arabidopsis

Figure 1. Performance comparisons between different regression models across four RNA-seq species: (a) Yeast, (b) B.subtilis, (c) Arabidopsis and (d) Mouse. Regression models compared here are random forest (RF) (i)model using all available transcription factors (TFs) as input, (ii)linear regression (ridge regression) using all TFs as input, (iii)RF model using gold standard (GS) edges for each target gene, (iv)linear model using GS edges, (v)RF model using top-k most important TFs inferred from model (i) where k is the number of TF edges in GS, (vi) RF model using minimal TF set deduced following algorithm 1. Each model's performance is measured as normalized RMSE relative to the target gene's standard deviation of expression. The results are presented as area under the curve (AUC) plot of normalized RMSE plotted against target gene's standard deviation of expression. From the figures we can see that a RF model utilizing all TFs as input features has the best predicting power in the regression task, where as regression based on existing GS edges tends to yield worse results.
